# Use of passive diffusion sampling method for defining NO_2 _concentrations gradient in São Paulo, Brazil

**DOI:** 10.1186/1476-069X-5-19

**Published:** 2006-06-13

**Authors:** Agnes Soares da Silva, Maria Regina Cardoso, Kees Meliefste, Bert Brunekreef

**Affiliations:** 1 Institute for Risk Assessment Sciences – IRAS, Utrecht University, Yalelaan 2, 3508 TD, Utrecht, The Netherlands; 2 Faculty of Public Health, University of São Paulo, Health and Environment Information Programme – PROISA, Av. Dr Arnaldo, 715, 01246-904 – São Paulo, SP, Brazil; 3 Julius Center for Health Sciences and Primary Care, University Medical Center, PO Box 85500, 3508 GA Utrecht, The Netherlands

## Abstract

**Background:**

Air pollution in São Paulo is constantly being measured by the State of Sao Paulo Environmental Agency, however there is no information on the variation between places with different traffic densities. This study was intended to identify a gradient of exposure to traffic-related air pollution within different areas in São Paulo to provide information for future epidemiological studies.

**Methods:**

We measured NO_2 _using Palmes' diffusion tubes in 36 sites on streets chosen to be representative of different road types and traffic densities in São Paulo in two one-week periods (July and August 2000). In each study period, two tubes were installed in each site, and two additional tubes were installed in 10 control sites.

**Results:**

Average NO_2 _concentrations were related to traffic density, observed on the spot, to number of vehicles counted, and to traffic density strata defined by the city Traffic Engineering Company (CET). Average NO_2_concentrations were 63μg/m^3 ^and 49μg/m^3 ^in the first and second periods, respectively. Dividing the sites by the observed traffic density, we found: heavy traffic (n = 17): 64μg/m^3 ^(95% CI: 59μg/m^3 ^– 68μg/m^3^); local traffic (n = 16): 48μg/m^3 ^(95% CI: 44μg/m^3 ^– 52μg/m^3^) (*p *< 0.001).

**Conclusion:**

The differences in NO_2 _levels between heavy and local traffic sites are large enough to suggest the use of a more refined classification of exposure in epidemiological studies in the city. Number of vehicles counted, traffic density observed on the spot and traffic density strata defined by the CET might be used as a proxy for traffic exposure in São Paulo when more accurate measurements are not available.

## Background

The combustion of fossil fuels in motor vehicles is one of the major sources of anthropogenic nitrogen oxides in most large cities worldwide. NO_2 _concentrations in urban spaces show a good correlation with traffic density [1-3] at the measured site, being its total concentration given by the actual local sources and by the urban and regional background [3]. The use of NO_2 _as a marker for traffic pollution may, however, hide complex chemical processes that occur in the atmosphere. Most of the NO_2 _is emitted as NO, which will be oxidised into NO_2 _mainly due to the presence of ozone [3]. Conversely, NO_2 _and other nitrogen oxides also contribute to the generation of ozone and other oxidant pollutants and are a precursor for the formation of nitric acid and, subsequently, the nitrate component of particulate matter (PM). Thus, NO_2 _is both a pollutant of concern and a surrogate for other concerns [4]. There are limitations for the use of NO_2 _as indicator of traffic-related air pollution because the mixture of organic and inorganic aerosols, oxidants and particles may vary in place and time, but the same would happen when using any of the other components [4].

There is a growing body of evidence about the relationship between exposure to high traffic density and the increase in respiratory symptoms [1,5-10]. Although no clear effect mechanism in the natural history of asthma and other allergic diseases has been documented, living close to busy roads has been revealed to enhance the demand for paediatric health care facilities and increase the use of medication [11-14]. It appears unlikely that a long-term exposure to pollutants or irritants alone is responsible for the secular increase in asthma and allergy observed in Western countries, but to what extent exposure to traffic pollution increases the risk for acute asthma or its inception remains to be clarified. Keil et al. (1996) [8], using the ISAAC questionnaire to assess the relationship between traffic density and wheezing and allergic rhinitis in children 12–15 years of age in Germany, found a positive correlation between the intensity of truck traffic and the prevalence of wheezing as well as allergic rhinitis. Exposure to traffic fumes has been associated with respiratory symptoms, especially wheezing and allergic rhinitis [6,7], but also with asthma [15,16], diseases of the lower respiratory tract [11], and reduced lung function [17].

In São Paulo, 90% of the air pollution in the inner city derives from motor vehicles [18]. In 2000 there were 3,790,475 vehicles [19] divided into cars, motorcycles, lorries and buses in the city of São Paulo, a large part of them older than ten years and without adequate pollution control, in a city of 10,435,546 inhabitants [20] in an area of 1,509 Km^2 ^[21]. Vehicle emissions are responsible for 98% of CO, 97% of hydrocarbons, 96% of NO_x _67% of SO_x _and 40% of the inhaled particulate matter present in the atmosphere of the city [18]. From the total of 14,000 Km of streets and avenues, only 98 Km have special lines for buses and the city Traffic Engineering Company (CET) monitors with traffic counting a maximum of 3,000 Km.

The city has a high traffic density due to the concentration of shopping centres, universities, schools, offices, banks, and industries. There are daily traffic jams in various points of the city, in spite of the restrictions for lorry circulation in many of the most important streets and avenues. Many initiatives were taken to minimise the problem, but the initial positive impact was shortly after overwhelmed by the increase of the fleet. Since 1996, a system has been placed that requires cars to take turns staying off the roads. This program, known as 'Rodízio', is enforced only within the central area, during peak hours on week days. The cars rotate based on the last number of their licence plate and there are heavy fines for offenders. Two numbers a day cannot circulate during the rush hours. This system affects 53% of the 24.5 million motorized trips a day made by private cars; the other 47% are made by public transportation [22]. A significant number of the fleet uses ethanol as fuel, although the quality of the emissions varies according to the age of the vehicle. Furthermore, ethanol is added to gasoline in a proportion in the blend of approximately 22% ethanol and 78% gasoline, which also contributes to reduce vehicles emission of aerosols and particulate matter.

Air pollution in São Paulo is being constantly measured by the State Environmental Agency, mainly by fixed monitoring stations, but there is no detailed information on the variation between places with different traffic densities. However, primary pollutants emissions from motor vehicles, such as elemental carbon, carbon monoxide (CO), sulphur dioxide (SO_2_), nitric oxide, and to a lesser extent nitrogen dioxide (NO_2_) show wide spatial variability across and between cities [23,24]. Secondary pollutants may be more evenly distributed, with the exception of ozone that might be low close to traffic emissions, because of the molecule's rapid destruction by short-lived nitric oxide (NO) [25].

Interpolated ambient concentrations based on measurements collected by the air quality monitoring network may be used in epidemiological studies, but spatial average ambient concentrations of primary emissions could be far less reliable when estimating the differences of individual exposure. Although the urban pollution mix related to motor vehicle emissions could be based on a myriad of inputs and assumptions such as fleet characterization, vehicle starts and stops, driving speeds, deterioration rates of pollution control systems, and other factors, no study had characterised a gradient of exposure in the urban space of São Paulo. There are different models for calculation of concentrations of exhaust gases in inner city streets of different configurations. This study was intended to identify a gradient of exposure to traffic-related air pollution in São Paulo for future applications in such refined models of dispersion, and to be used eventually in studies on adverse health effects of traffic pollution.

## Methods

Palmes tubes [26], a passive sampler consisting of a small cylindrical tube with a metal grid of stainless steel coated with TEA (tri-ethanol amine) at the bottom of the tube, were used to measure ambient NO_2_. NO_2 _was determined after extraction using a spectrophotometric Saltzman's reaction of ion chromatography. The concentration of NO_2 _was calculated based on the Fick's law using the recorded sampling time, the physical characteristics of the tube and the diffusion coefficient.

Measurements of NO_2 _were conducted in 36 different locations on streets, roads and avenues, chosen to be representative of road types and traffic densities in São Paulo, in two one-week periods. From these 36 sites, the Palmes tubes could be recovered in 34 sites in the first period and 35 sites in the second period, but only in 33 sites could they be recovered in both periods. The statistical analysis was performed using the results of these 33 sites.

The Palmes tubes were prepared by the laboratory of the Institute for Risk Assessment Sciences – IRAS, University of Utrecht, the Netherlands, and they were analysed by the laboratory of microbiology and chemistry of the Faculty of Public Health, University of São Paulo (FSP/USP), São Paulo. Control samples were analysed by the laboratory of the IRAS. The protocol for preparation, measurements and analysis was similar to the collaborative study on the impact of Traffic-Related Air Pollution on Childhood Asthma – TRAPCA (EU Environment project PL970969).

### Field procedures

The two one-week samples were taken during winter in São Paulo, when the highest pollution is expected due to thermal inversion that frequently occurs during this season. The first period was from July 18–19 to 25–26 and the second in August, from 10–11 to 17–18, both in the year 2000. To avoid meteorological influences, all sites were visited within a maximum of 48 hours difference in each of the periods. The chosen sites were representative of the most common types of streets in Sao Paulo, varying from large avenues to small canyon type streets; and from busy roads to suburban local streets.

The sampling frame was installed in such way that the Palmes tubes were permanently in a vertical position with the inlet facing down when inserted in the frame and the drip line of trees was avoided in all cases. The metal frame was large enough to protect the tubes and to maintain them in a fixed position. There were no restrictions of airflow around the sampler. The sampler was fixed about one meter from the wall and distant from any other obstacle of air flow. It was also placed distant from exhaust flues or vents and at a height of at least 3 meters above the ground to prevent vandalism.

Sampling started with the removal of the lid from the tube and placing it in the frame and ended by placing the small lid on the tube and removing the tubes from the sampling frame. The time was recorded in a field form in the beginning and at the end of the period, to the nearest 5 minutes.

### Storage

After sampling, tubes were stored in the refrigerator at 4 ± 3°C in the dark in the FSP/USP until they were analysed. In São Paulo the tubes were analysed within two weeks and the control samples, in The Netherlands, within two months.

### Laboratory analysis

Standard procedures were adopted to assure quality of the measurements and acceptable within samples variability. Periodically, check samples were analysed to detect potential problems with the reagents or the spectrophotometer. No check samples should deviate more than 10% from the amount of nitrite of the matching calibration standards.

### Quality control

Each site had duplicate measurements. Contradictory measurements (duplicates that differed more than 30% from each other) were rejected. Inter-laboratory quality control was achieved by taking another set of duplicate samples at 10 sites for analysis by the Laboratory of the IRAS group in the Netherlands. The Palmes tubes from 8 of these sites were recovered in both, first and second periods of measurements.

The coefficients of variation between duplicates analysed in São Paulo and at IRAS were within the acceptable range. Within and between laboratory reliability were checked, using intra-class correlation coefficient as described by Szklo & Javier Nieto [27]. The intra-class correlation coefficient for the tubes analysed in São Paulo was 0.82 (95% CI: 0.70 – 0.93) in the first sampling period and 0.89 (95% CI: 0.82 – 0.96) in the second. When the two laboratories (FSP/USP and IRAS) were compared, the intra-class correlation coefficients were 0.94 (95% CI: 0.87 – 1.00) and 0.82 (95% CI: 0.62 – 1.00) for the first and second sampling periods, respectively.

Data from three fixed monitoring stations of CETESB used as an external control also showed differences on the NO_2 _mean concentration between the two sampling periods, in the same range as those measured by the Palmes tubes.

### Traffic density

With the objective of finding reliable proxy measures for traffic exposure in São Paulo, the streets were evaluated with regard to the traffic density using three different criteria: (1) direct observation by the fieldworkers: the flow of vehicles was observed during fieldwork and the streets were classified into two categories – local (low) and heavy traffic density; (2) CET classification: using the CET official system, the streets were grouped into three categories – low, intermediate and high traffic density; and (3) number of vehicles counted: using the CET data as a continuous variable (log transformed).

### Data analysis

Data from the field forms and from laboratory results were recorded using Excel. Statistical analysis was performed using the software SAS^® ^8.2. Tests used included correlation between number of vehicles (log) and NO_2 _concentration; multiple linear regression models were used for the different measures of traffic density; logistic regression models were used for traffic counting and NO_2 _concentration; one-way ANOVA procedure, was used to study the relationship between daily wind speed in the first and second period and average daily NO_2 _concentration measured by the CETESB; intra-class correlation tests were used for quality control between the double Palmes tubes in each site and between the tubes analysed in Sao Paulo and at IRAS.

## Results

**Figure 1:**
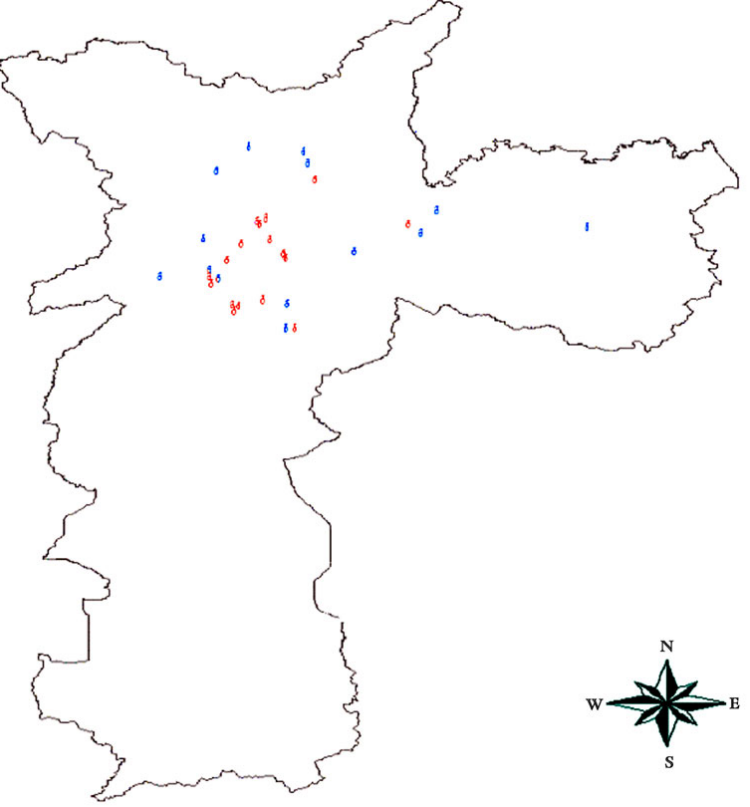
Distribution of the passive samplers sites in different regions of the City of São Paulo.

The 33 observation points were distributed in all regions of the inner City of São Paulo (Figure 1). The sites were also chosen to represent the variety of types of streets in the city.

**Figure 2:**
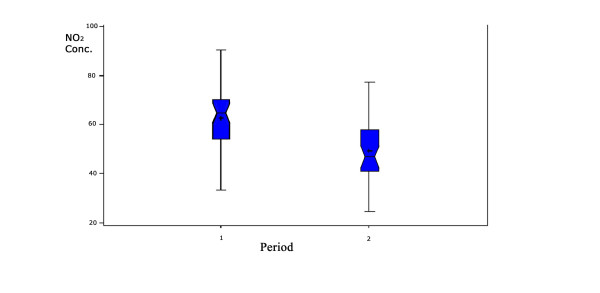
**Mean NO_2 _levels in the first and second periods of measurement**. (*p = 0.05*) Period 1 = July; Period 2 = August.

**Figure 3:**
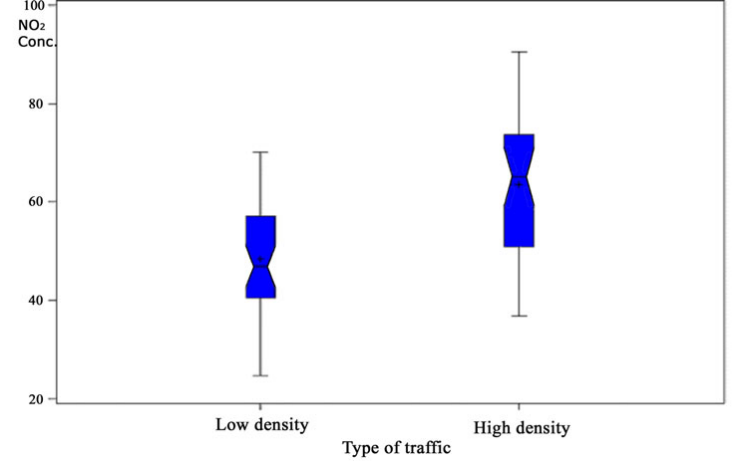
**NO_2 _concentration according to traffic density using on-site direct observation**. *p *= 0.005.

The overall average concentrations of NO_2 _were 63μg/m^3 ^(95% CI: 57μg/m^3 ^– 67μg/m^3^) and 49μg/m^3 ^(95% CI: 45μg/m^3 ^– 54μg/m^3^) in the first and second sampling periods, respectively. When the sites were classified according to the direct observation of traffic density, we found the following NO_2 _concentrations: heavy traffic (n = 17) 64μg/m^3 ^(95% CI: 59μg/m^3 ^– 68μg/m^3^); local (low) traffic (n = 16): 48μg/m^3 ^(95% CI: 44μg/m^3 ^– 52μg/m^3^) (*p *< 0.001). Sites classified as local traffic (n = 16) had a median of 81 and a mean of 95 vehicles/per hour (95% CI: 68 – 122; min = 0; max = 267); sites classified as heavy traffic had a median of 2,742 and mean of 2,892 (95% CI: 2,346 – 3,437; min = 556; max = 6,354). Figure 2 shows that the mean levels of NO_2 _in the periods 1 (July) and 2 (August) differs significantly (*p *< 0.001), being almost at the same range of variation when comparing sites with different observed traffic densities as shown in Figure 3. These differences must additionally account for the fact that the traffic peak flow is lower in July than in August, specially in the morning, due to school holidays [28].

**Figure 4:**
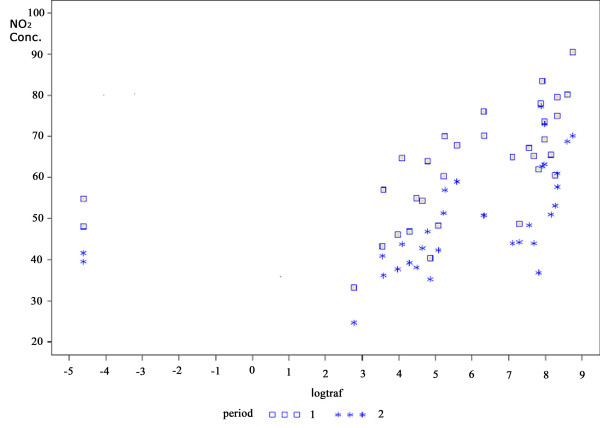
**NO_2 _concentration according to traffic counting (log)**. *r *= 0.65 *p *< 0.001 for periods 1 + 2. *r *= 0.75 *p *< 0.001 for period 1. *r *= 0.71 *p *< 0.001 for period 2.

**Figure 5:**
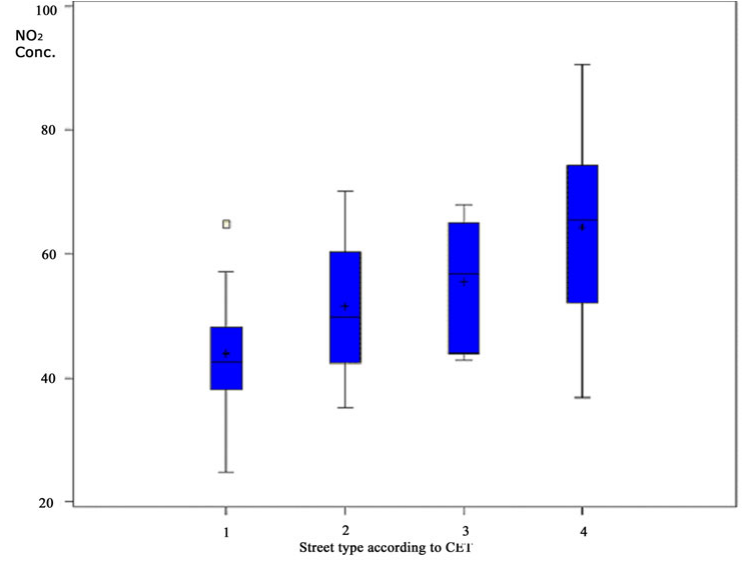
**NO_2 _concentration according to the CET street classification**. **1 **= LOC – Streets only with local traffic, low traffic density. **2 **= COL – Local and intermediate traffic, predominance of local traffic and median flow of vehicles. **3 **= ART – Local and intermediate traffic, and median to high flow of vehicles. **4 **= VTR – Avenues that collect all traffic and give access to different destinations and have high traffic density.

The correlation between NO_2 _concentration and the log of vehicles counting per hour adjusted for the effect of period is shown in Figure 4. Figure 5 shows that the classification of the streets used by the CET also has a positive correlation with NO_2 _concentration. Detailed analysis using CET classification was not possible due to the small number of sites in the groups 2 and 3 (Collector and Arterial types of street). When these two groups were combined, the positive correlation was maintained, showing a difference of exposure between the sites in the order of 10μg/m^3 ^NO_2_, being around 20μg/m^3 ^between LOC (low traffic sites) and VTR (high traffic density) sites. When the classification Local and Heavy traffic, given by direct observation, was used, the data collected showed similar results, i.e. the difference between the levels of NO_2 _was in the order of 20μg/m^3^.

Although the differences between the first and the second periods of measurements are in the same range, in both periods the differences between the sites remain practically the same, confirming that the classification by direct observation is strong enough to show differences in exposure, as seen in Table 1.

**Table 1:** NO_2 _concentration according to on-site traffic density classified by direct observation and period of measurement

**Traffic type**	**Period**	**Mean of NO**_2_**conc μg/m**^3^	**Std. Dev. of NO**_2_**conc**
Low traffic density	1	53	10
Low traffic density	2	42	8
High traffic density	1	71	10
High traffic density	2	56	12

Table 2 shows the results of the regression analyses using the different measures of traffic density. The number of vehicles counting alone explained 24% of the variation of the NO_2 _concentration, the observed traffic density 30% whilst the CET classification explained 35% (*p *< 0.0001).

**Table 2:** Regression analysis of NO_2 _concentration and traffic counting (log)

**NO_2 _concentration**	**Coef**	**Std Err.**	**t**	**p > |t|**	**95% Conf. Interval**
Period	-13.10	2.40	-5.51	0.000	-17.80 – – 8.30
Log traffic	0.35	0.61	0.57	0.570	-0.88 – 1.58
Low	(reference)				
Intermediate	7.90	3.90	2.03	0.047	0.11 – 15.70
High	18.30	4.60	3.99	0.000	9.10 – 27.50
Constant	62.90	4.40	14.32	0.000	54.10 – 71.70

R-squared = 0.58.

*adjusted for period and the CET street classification

Table 3 shows the results of the multiple linear regression models for the different measures of traffic density controlling for period. In these analyses, the number of vehicles counting explained 44% of the variation of the NO_2 _concentration, the observed traffic density 50% whilst the CET classification explained 56% (*p *< 0.0001).

**Table 3:** Multivariate analysis of NO_2 _concentration for different measures of traffic density adjusted for period

**Variables**	**Coef.**	**95% Conf. Interval**	**Adj R^2^**
Number of vehicles counting (log)	2.29 *	1.45 – 3.14	0.44
Observed traffic density	15.90 *	10.90 – 20.90	0.50
CET classification			0.56
- Intermediate	9.10 *	2.50 – 15.70	
- High	20.40 *	14.70 – 26.00	

* p < 0.0001

**Figure 6:**
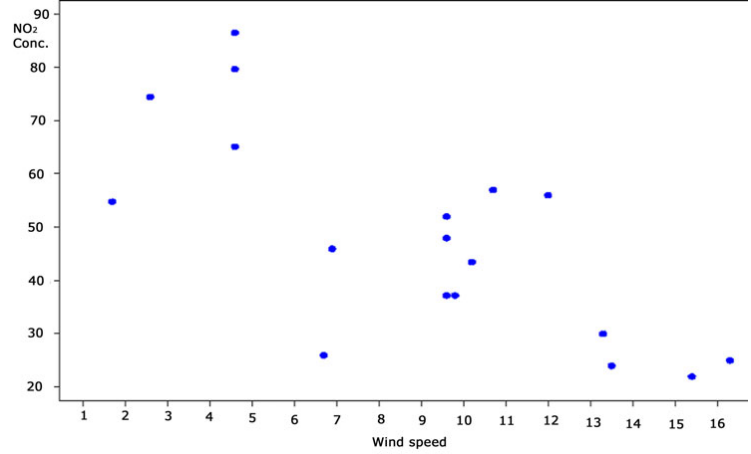
Daily mean NO_2 _concentration* and wind speed** in the first and second periods. *Source: CETESB. **Source: IAG – Instituto de Agronomia, Geofísica e Ciências Atmosféricas. *r *= 0.63 *p *= 0.05.

In order to identify possible causes for the differences in the NO_2 _concentrations in the first and second periods, daily temperature (min, max and mean), wind speed (mean, min and max), daily mean humidity and rainy days were compared. Although the number of sites and the time used for the analysis do not allow firm conclusions, the daily average wind speed showed a negative correlation with daily mean concentration of NO_2 _at the stations measured by the CETESB as shown in Figure 6 (*r *= -0.63, *p *= 0.05). Using the one-way ANOVA procedure, the differences between wind speed in the first and second periods were found to be significant (*p *= 0.002), which could partially explain the differences of NO_2 _concentration observed in the two periods (not shown).

## Discussion

The levels of NO_2 _measured in the two periods show that both low and high traffic density sites presented NO_2 _concentration levels above 40μg/m^3^, which is the WHO Air Quality Guideline for annual average NO_2 _concentrations [29]. The Palmes tubes have been extensively used before to measure NO_2 _concentration for health effect studies. In the Netherlands, two exposure studies used the Palmes tubes to measure NO_2 _concentration to assess exposure of children to air pollution from traffic, Janssen et al. [30] measured PM_2.5_, NO_2 _and benzene in and outside 24 schools located within 400 m of motorways, and Roorda Knape et al. [1,31] measured PM_10_, PM_2.5_, benzene, NO_2 _and black smoke in six residential areas with homes located within 300 m from a major motorway. Kramer et al. [9] used traffic counting, outdoor and personal monitors to study exposure in three areas of Dusseldorf, two urban and one suburban. Mean NO_2 _outdoors levels measured in one-week period with Palmes tubes were 62μg/m^3 ^and 57μg/m^3 ^for the urban areas, and 44μg/m^3 ^for the suburban area, similar to those found in São Paulo. Mean levels of outdoor NO_2 _varied significantly between the two studied sites, being 48μg/m^3 ^and 45μg/m^3 ^close to the highway and 31μg/m^3 ^(*p *< 0,01) and 32μg/m^3 ^(*p *< 0.05) circa 300 m further. For the SAVIAH study, Pikhart et al. [32] measured NO_2 _in three two-weeks period using Palmes tubes in 80 sites in two districts of Prague, finding a median NO_2 _concentration (25th and 75th percentiles) of 36μg/m^3 ^(28μg/m^3 ^– 45μg/m^3^). Gehring et al. [5], reporting the TRAPCA study in Germany, estimated the long-term exposure to NO_2 _in the range of 11.9–21.9μg/m^3 ^and found that NO_2 _levels were mostly influenced by traffic intensities until 250 m of highways and address density at 300 m. Raaschou et al. [33], used front-door concentrations of traffic exhaust fumes for classifying children into two exposure groups in Copenhagen, in urban and rural areas. Actual exposure status was obtained from personal exposure measurements of NO_2 _that were compared to NO_2_measured at the front door of the houses, accounting for or excluding children with known indoor exposure. The study concluded that NO_2 _at the front door could be used to classify exposure of the children as a marker of traffic pollution because traffic is the dominant source of NO_2_in the streets, but not in the rural districts, where the results cannot be assigned to any specific local source of outdoor NO_2 _pollution.

The study done in São Paulo suggests that there is a gradient of NO_2 _concentration in accordance with different traffic densities. The differences found were large enough to support health impact studies considering different levels of exposure using surrogates variables such as observed traffic density, counting of the number of vehicles, and the local traffic engineering classification system.

## Conclusion

The difference of NO_2 _level between local and heavy traffic sites is strong enough to distinguish levels of exposure in the city of São Paulo. This new information should be considered when planning future epidemiological studies in São Paulo and when analysing health effects related to traffic exposure.

Traffic counting, traffic density observed on-site, and traffic density strata defined by the CET correlate well with NO_2 _concentration of the outdoor air in São Paulo City, which suggests that they could be used as proxy for traffic exposure, when more accurate measurements are not available. These findings are well in accordance with current literature.

This information is particularly useful for developing countries, where feasibility of environmental measurements might be limited by availability of technical resources and financial capability.

## Abbreviations

ANOVA – Analysis of Variance between groups

CET – Companhia de Engenharia de Trafego (Traffic Engineering Company)

CETESB – Companhia de Tecnologia de Saneamento Ambiental (State of Sao Paulo Environmental Agency)

CO – Carbon Monoxide

CI – Confidence Interval

FSP/USP – Faculdade de Saúde Pública da USP/Universidade de São Paulo (Faculty of Public Health/University of São Paulo)

IRAS/UU – Institute for Risk Assessment Sciences, University of Utrecht, The Netherlands

ISAAC – International Study of Asthma and Allergies in Childhood

NO – Nitric oxide

NO_2 _– Nitrogen dioxide

NO_x _– Nitrogen oxides

PM – Particulate Matter

PROISA – Programa de Informação em Saúde e Ambiente (Health and Environment Information Programme)

SO_x _– Sulphur Oxides

TEA (tri-ethanol amine)

## Competing interests

The author(s) declare that there are no competing interests.

## Authors' contributions

ASdS contributed to the design of the study, prepared the Palmes tubes under supervision of KCM, executed the field work, analysed the data and wrote the first draft and the final version of the manuscript. MRC organized the field work, contributed to the analysis and interpretation of the data, reviewed the manuscript and made relevant suggestions for both the discussion and conclusion. BB contributed to the study design and to the analysis and interpretation of the data, suggested references, and made relevant contributions to the discussion and conclusions. KCM contributed greatly to the design of the study, supervised the preparation of the Palmes tubes and performed the tubes pre- and pos-exposure quality control check in the Netherlands.
